# Essential roles of EphrinB2 in mammalian heart: from development to diseases

**DOI:** 10.1186/s12964-019-0337-3

**Published:** 2019-03-25

**Authors:** Sheng-an Su, Yao Xie, Yuhao Zhang, Yutao Xi, Jie Cheng, Meixiang Xiang

**Affiliations:** 1 Department of Cardiology, The Second Affiliated Hospital, Zhejiang University School of Medicine, Hangzhou, 310009 China; 2 Texas Heart Institute, Houston, 77030 USA

**Keywords:** EphrinB2, Bidirectional signaling, Development, Cardiac remodeling, Therapeutic molecules

## Abstract

EphrinB2, a membrane-tethered ligand preferentially binding to its receptor EphB4, is ubiquitously expressed in all mammals. Through the particular bidirectional signaling, EphrinB2 plays a critical role during the development of cardiovascular system, postnatal angiogenesis physiologically and pathologically, and cardiac remodeling after injuries as an emerging role. This review highlights the pivotal involvement of EphrinB2 in heart, from developmental cardiogenesis to pathological cardiac remodeling process. Further potential translational therapies will be discussed in targeting EphrinB2 signaling, to better understand the prevention and treatment of cardiovascular diseases.

## Background

Ephrins (erythropoietin-producing hepatocellular receptor interacting proteins) are membrane-tethered ligands for Eph tyrosine kinase receptors (erythropoietin-producing hatocellular receptors), ubiquitously expressed in mammal. According to the ways of anchoring cellular membrane, Ephrins were divided into type A and type B subfamilies. All five members of EphrinAs connected the extracellular Eph-binding domain to the transmembrane segment by utilizing a glycosyl phosphatidylinositol (GPI) anchor. Whereas, all three members of EphrinBs employed a single-pass transmembrane region to fuse the Eph-binding globular domain with a cytoplasmic tail followed by several phosphorylation sites and PDZ domains [1]. Classic mode of Ephrin/Eph signaling was bi-directional, involving Ephrin binding *in trans* to Eph which activated the receptor’s forward signaling, as well as Eph receptor eliciting Ephrin *in trans* which activated the ligand’s reverse signaling. Noteworthy, Ephrin reverse signaling, in certain circumstances, could be activated independently of the associated Eph receptors [2–4].

The interaction between Ephrin and Eph was critical in development and a variety of diseases. EphrinBs have been evidenced essential in a serious of developmental processes including vasculogenesis, neural and cardiac development. Meanwhile, EphrinBs were physiologically involved in regulating synaptic plasticity, axonal guidance and maintaining vascular homeostasis [5, 6]. Pathologically, EphrinBs were implicated in cancer progression and neurodegenerative diseases [7, 8]. Among the EphrinBs family, EphrinB2 and its preferentially paired receptor EphB4 played crucial roles during the development of cardiovascular system and postnatal angiogenesis. Beyond angiogenesis, EphrinB2 was emerging as an important regulator in cardiac remodeling after injuries, implying a potential therapeutic target in cardiovascular diseases. Thus, in this review, we provide an overview on the recent knowledge gained on the impact and function of EphrinB2 in heart from development to diseases. We also updated the potential therapeutic strategies that interfere with EphrinB2 signaling.

### EphrinB2-mediated bi-directional signaling

**Fig. 1:**
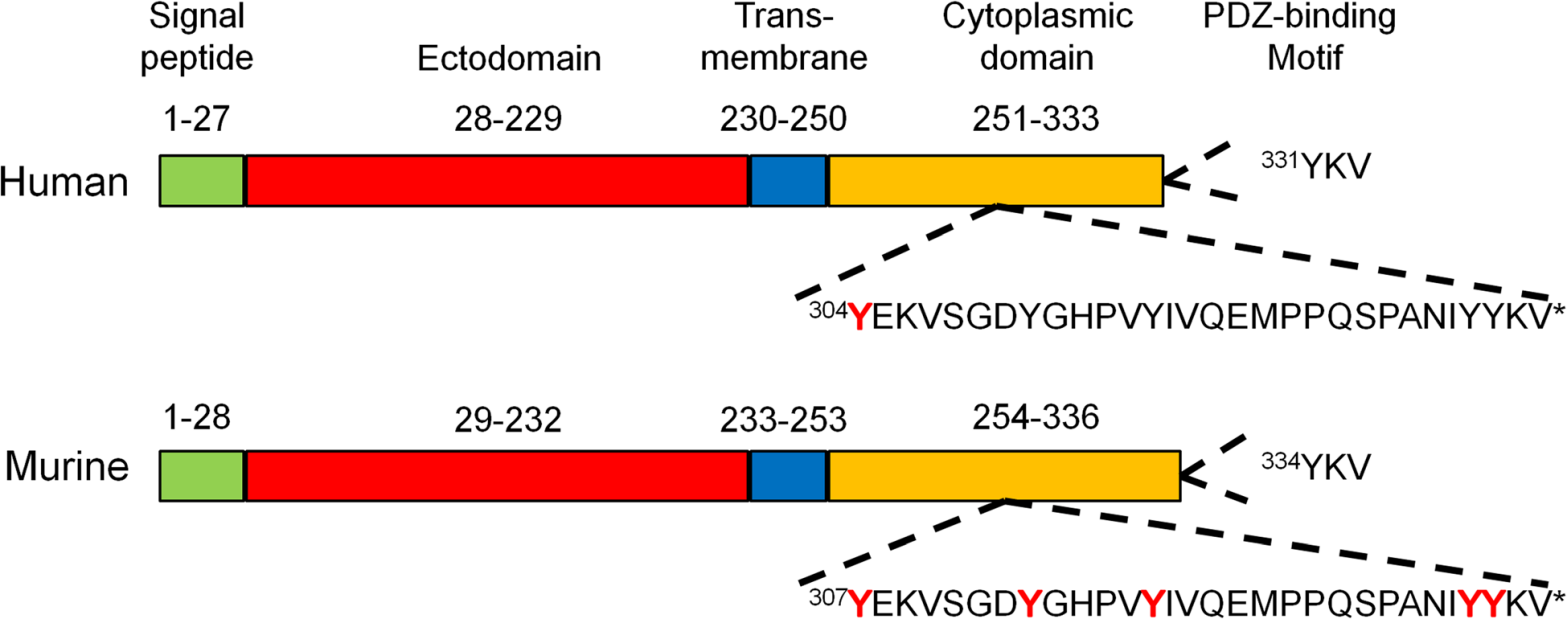
Domain structure of EphrinB2 in human and murine. The conserved tyrosine phosphorylation sites identified were labeled in red, while Tyr304 in human linked phosphorylation signaling with PDZ-dependent signaling. The last three amino acids composed a PDZ-binding motif in both human and murine

**Fig. 2:**
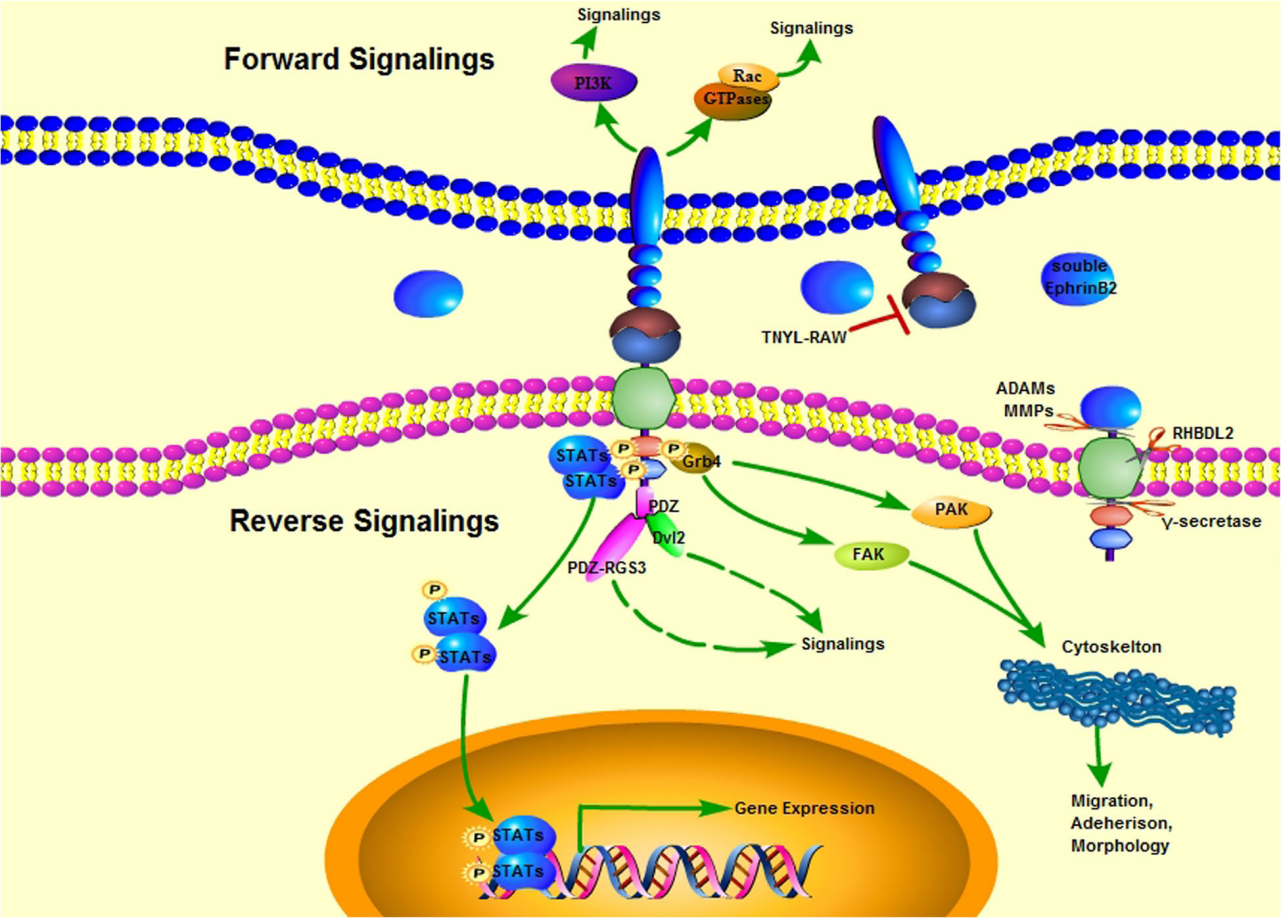
Schematic diagram of EphrinB2-mediated bidirectional signaling and potential therapeutic molecules targeting EphrinB2

EphrinB2 was a highly conserved protein in mammal. Although human EphrinB2 was constituted 333 amino acids, while murine EphrinB2 had three more amino acids. They shared over 98% homology of amino acid sequence and roughly same structure. The domain structure of EphrinB2 consisted of ectodomain (1–229 aa in human, 1–232 aa in murine), transmembrane domain (230–250 aa in human, 233–253 aa in murine) and cytoplasmic domain (251–333 aa in human, 254–336 aa in murine), while the last three amino acids consisted a PDZ-binding motif in both human and murine [9] (Fig. 1). The forward signals originating from EphrinB2-binding Eph receptors mainly included the activation of PI3K and Rac-GTPases pathways [10]. The reverse signaling was transduced by a selfphosphorylation-dependent way and a PDZ-dependent way respectively due to the lack of intrinsic catalytic activity in EphrinB2. The tyrosine phosphorylation of EphrinB2 mediated by Src kinases [11] led to a recruitment of the SH2/SH3 domain-containing adaptor proteins such as Grb4 [12, 13], Stat1 [14] and Stat3 [15]. Grb4 regulated a serious of cytoskeleton signaling including FAK, PAK pathways affecting cell adhesion and migration [12], while Stat1 and Stat3, as critical transcription factors, transduced EphrinB2 signaling from cell membrane to nucleus. However, the exact phosphorylation sites of EphrinB2 remained obscure. The PDZ-dependent signaling was activated by binding proteins with PDZ domain to the PDZ motif, such as PDZ-RGS3 [16] and Dvl2 [17, 18], playing an important role in EphrinB2-mediated embryonic development and angiogenesis despite of the unclarified downstream efforts (Fig. 2). Notably, the crosstalk between phosphorylation signaling and PDZ signaling was existed. Phosphorylation of Tyr304 in human EphrinB2 conferred high-affinity and bifunctional binding activity to both Grb4 and PDZ-RGS3 [13].

### EphrinB2 in heart development

EphrinB2 was of great importance in heart development. Conventional knockout of EphrinB2 was embryonic lethal in mice, which attributed to the defects of angiogenesis in yolk sac and myocardial trabeculation [19, 20]. Meanwhile, obvious arrest of heart development in these EphrinB2-null embryos was observed, including incompletion of cardiac looping, failure of endocardium expansion and formation of myocardial trabeculation. Afterwards, the endothelial specific knockout of EphrinB2 exhibited an indistinguishable angiogenic remodeling and cardiac defects from those of the global EphrinB2 knockout, further suggesting that EphrinB2 is affirmatively required in mouse embryonic endothelial and endocardial cells for proper cardiovascular development [21]. However, it was noteworthy, that whether EphrinB2 serves as a ligand or a receptor was still concealed. One study reported that the truncation of the majority of the EphrinB2 C-terminal cytoplasmic tail with or without HA tag (a loss-function approach of EphrinB2 reverse signaling) results in the similar embryonic lethality as the null, leading to a conclusion that EphrinB2 acts essentially as a receptor to transduce the reverse signaling in the early cardiac development [22]. In contrast, deletion of the same cytoplasmic residue but rather with βgal infusion (EphrinB2^ΔV-βgal^) allowed full embryonic development and birth of neonatal mice, in which EphrinB2-mutant protein preserved the surface binding activity and functioned as a proper ligand to stimulate EphB forward signaling [23]. Thus, the bidirectional effects of EphrinB2 may be both involved in embryonic cardiogenesis. To further elucidate the molecular mechanisms required for EphrinB2 transducing cellular signaling in vivo, two sets of knock-in mice were generated: (1) EphrinB2^ΔV^ mice, which was lacking the C-terminal PDZ interaction residue to disrupt PDZ-dependent EphrinB2 signaling and (2) EphrinB2^5F^ mice, which was lacking phosphotyrosine-dependent EphrinB2 signaling with inactive mutation in 5 conserved tyrosine sites. Both of the mice survived the requirement of EphrinB2 in embryonic angiogenesis, while EphrinB2^5F^ mice survived to adulthood but EphrinB2^ΔV^ mice died from failure of lymphatic vasculature maturation [17]. These data suggested that EphrinB2 reverse signaling may be complex and alternative in the embryo. Severe defects may only appear when both PDZ- and phosphorylation-dependent signalings are stripped off.

Additional data revealed that EphrinB2 reverse signaling is indispensable for cardiac valve maturation and the following neonatal survival. The EphrinB2^ΔV-βgal^ mice eventually died within first day after birth, exhibiting extremely thickened aortic and pulmonary valves [23]. A massive accumulation of mesenchymal cells around the aberrant cardiac valves implied that loss of EphrinB2 reverse signaling possibly disrupts the mesenchymal-to-epithelial transition in cardiac valve maturation, while the mechanism remained a further investigation.

The genetic evidences in mice have identified that the cellular location of EphrinB2 was not restricted in endothelial cells, but was also in mesenchymal cells, vascular smooth muscle cells and pericytes surrounding the angiogenic vessles [19, 20, 24, 25]. It was supposed that EphrinB2 bridges the communication between these support cells and endothelial cells, as well as inter-endothelial interactions [26]. Mechanistically, EphrinB2 controlled the internalization of VEGF receptors-VEGFR2 and VEGFR3 in endothelial cells through PDZ-dependent reverse signaling [27, 28]. Within the trafficking of VEGFR, EphrinB2 was critically involved in VEGF signaling and thus promoted developmental angiogenesis and lymphangiogenesis. Lately, a soluble EphrinB2 (sEphrinB2) which was the ectodomain of EphrinB2 cleaved by A Disintegrin and Metalloprotease (ADAM) 10 was identified in both embryonic development and adult fibrotic diseases in mice [29, 30]. The existence of sEphrinB2 implied a potential approach of EphrinB2 during the inter-endothelial interaction. Since EphB4 was also capable of modulating VEGFR2 signaling through phospho-Erk1/2 [31], it is speculative that there may exist both autocrine and paracrine forms of EphrinB2 to activate the bidirectional signalings during the crosstalk between endothelial cells and the support cells.

Similarly, Sonia et al. reported that in avian embryos, both EphrinB2 and B1 were expressed in epicardium and then restricted in perivascular fibroblasts of the atrioventricular sulcus during the development. These EphrinBs regulated the migration of epicardial cells in the early stage and later participated in the coronary vascular development via perivascular fibroblasts [32].

**Fig. 3:**
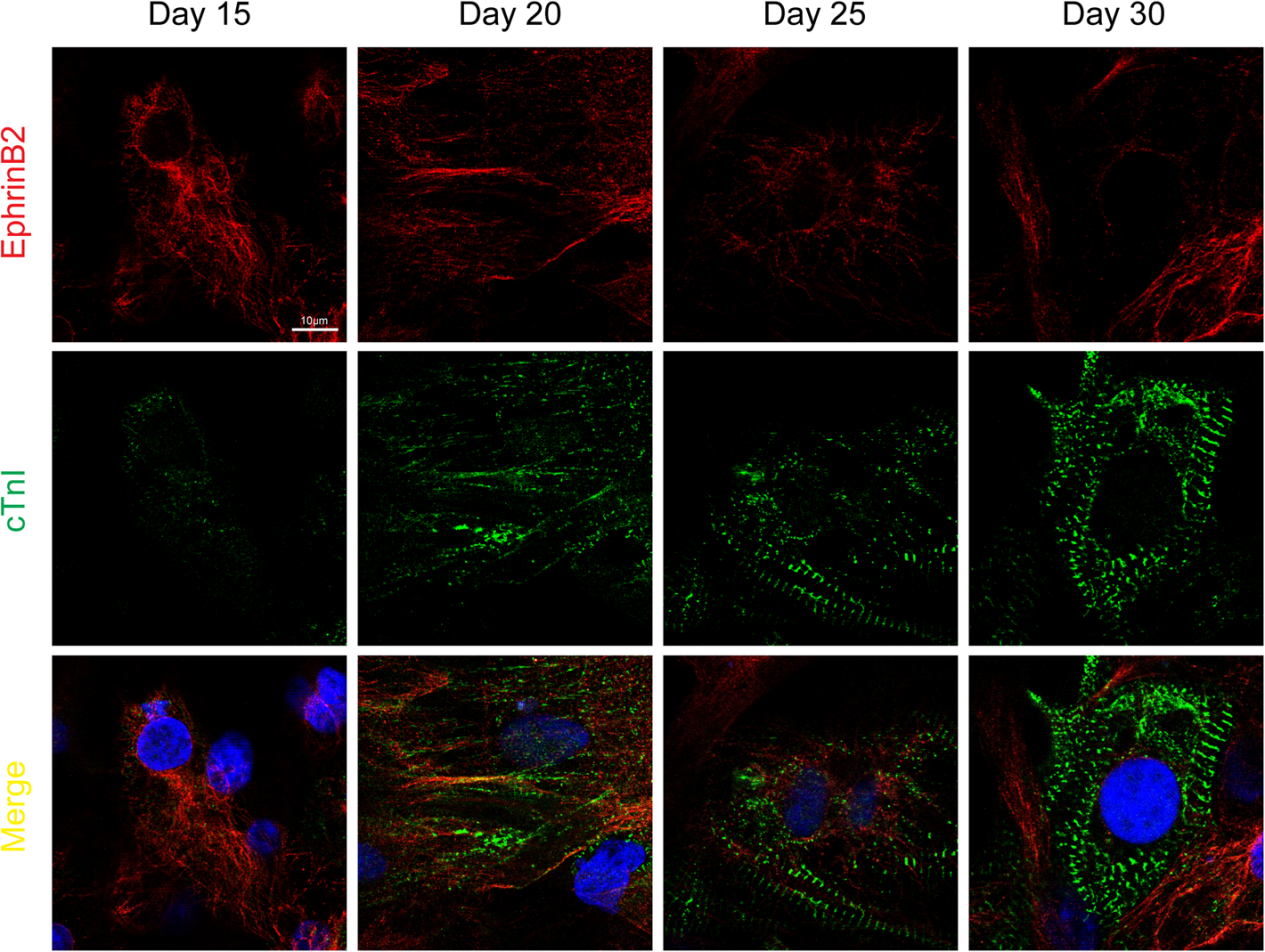
Labeling human induced pluripotent stem cells (hiPS)-differentiated cardiomyocytes undergoing maturation with EphrinB2 (red), Troponin I (green) and DAPI (blue) by immunofluorescence staining. Scale bar represents 10 μm

Recently, accumulating researches uncovered the role of EphrinB2 in cardiomyocyte differentiation. In-vivo genetic data showed that the EphrinB2, directly induced by Notch1/RBPJK signaling in primitive myocardial epithelium during embryonic cardiogenesis, guided the endocardial cells towards the cardiomyocyte differentiation, leading to the transition of primitive myocardial epithelium into myocardial trabeculation and ventricular chamber formation [33]. Consistently, in-vitro data confirmed that EphrinB2/EphB4 signaling is involved in cardiac lineage development. Disrupting the interaction of EphrinB2 and EphB4 by utilizing specific antagonist peptide during murine embryonic stem cell (ESC) differentiation impaired the ESC derived-cardiac progenitor cells stepping towards cardiomyocytes [34, 35]. Interestingly, the expression of EphrinB2 in cardiomyocyte showed a dynamic alteration from neonatal to postnatal. Abundant expression of EphrinB2 was detected in neonatal murine cardiomyocyte, while adult murine cardiomyocyte only expressed EphrinB2 transcripts but failed to be detected at protein level physiologically [24, 25, 36]. Meanwhile, EphrinB2 expressed in murine ESC-derived cardiac progenitors but not the differentiated cardiomyocytes [34]. By employing human induced pluripotent stem cells (hiPS), we discovered that in hiPS-differentiated cardiomyocytes, EphrinB2 remarkably decreased during cardiomyocyte maturation (Fig. 3). The immature cardiomyocytes including fetal and neonatal ones contained proliferating ability and regenerated even after extensive injuries, whereas adult cardiomyocyte were mostly post-mitotic with rare proliferating capacity [37]. Likely, the alteration of EphrinB2 in cardiomyocyte from immature to adult suggested a possible role of EphrinB2 in cardiomyocyte proliferation and even cardiac regeneration in the pathological conditions such as heart failure.

### EphrinB2 in heart diseases

#### EphrinBs in cardiac tissue architecture

In adult mouse heart ventricles, the EphrinB ligands are preferentially expressed in the vasculature and EphB receptors in cardiomyocytes [36]. Cardiomyocyte EphB4 which was activated by EphrinB2-Fc resulted in a significant inhibition of gap junctional intracellular communication between cardiomyocytes, suggesting that EphrinB/EphB signaling could modulate the electrical coupling of cardiomyocytes via gap junctions [36]. Noteworthy, another member of EphrinB family-EphrinB1 was discovered to be expressed in the lateral membrane of mouse cardiomyocyte, serving as a novel specific component to maintain the stability of cardiac tissue architecture [4]. Further investigations were urgently required to explain how EphrinB/EphB signaling functioned in cell-cell contraction to stabilize the cardiac architecture.

#### EphrinB2 in angiogenesis after cardiac injuries

Revascularization could therapeutically recover the injured myocardium in ischemic cardiovascular diseases such as myocardial infarction (MI) and ischemia/reperfusion (I/R) injury. Both the expression of EphrinB2 and EphB4 dramatically increased in MI, Brain IR injury and limb ischemic models in murine [38–41], suggesting the process of neovascularization occurred. Since EphrinB2/EphB4 controlled the endothelial spouting angiogenesis, which is essential to form a functional vessel [42], a certain activation of EphrinB2/EphB4 signaling in murine has been proved to induce angiogenesis in ischemic myocardium and brain where new blood vessels were urgently required [38, 40]. However, excessive expression of EphrinB2 in non-infarcted myocardium or physiologically-cultured endothelial cells did not work with few morphological alterations [40, 43]. Moreover, the mammalian inflammatory cells including neutrophil [44–46], macrophage [47] and T cell [48], all of which were evidenced to express EphrinB2, displayed a pro-angiogenic capacity during inflammatory angiogenesis which was beneficial for ischemic heart diseases. By using EphrinB2-Fc stimulation, Broqueres et al. have proved that the EphrinB2-activated peripheral blood mononuclear cells from diabetic patients could restore the impairment of postischemic neovascularization [49]. Thus, proper pathological/physiological stimulus seemed necessary to activate EphrinB2-regulated revascularization after cardiac injuries, whereas the conditions still need to be optimized and the mechanism was far from being resolved.

#### EphrinB2 in cardiac fibrosis

As expanded above, EphrinB2 was also expressed in mesenchymal cells, especially high in fibroblast. A decade before, pericyte-EphrinB2 knockout mice were observed with massive fibrosis surrounding aberrant vessels in skin [50], implying a potential role of EphrinB2 in organ fibrogenesis. Afterwards, EphrinB2 was proved to be involved in the fibrotic process of skin [51], retina [52] and kidney [53], respectively. Lately, two studies clarified the role and mechanism of EphrinB2 involved in lung and cardiac fibrosis. Although EphrinB2 emerged as a potent pro-fibrotic factor in the both organs after injury, EphrinB2 contributed to cardiac fibrosis through a phosphorylation-dependent signaling, leading to the transition of cardiac fibroblast to myofibroblast [15], while in lung fibrosis, fibroblast-derived EphrinB2 was cleaved as a soluble type to activate EphB forward signaling in an autocrine way [29]. Cardiac fibrosis, triggered by cardiac ischemia, hypoxia, overload-pressure, etc., was a critical pathological remodeling process after cardiac injury. EphrinB2 enhanced the TGF-β (Transforming Growth Factor-β) /Smad3 signaling-mediated cardiac fibrosis through regulating the interaction between Stat3 and Smad3 [15]. Interestingly, TGF-β also induced EphrinB2 expression in fibroblast, suggesting a positive feedback between EphrinB2 and TGF-β signaling [54]. Meanwhile, similar activation of EphrinB2 was observed in endothelial cells within TGF-β stimulation [55]. TGF-β-induced phenotype switch of endothelial cells towards mesenchymal cells was one of the mechanisms contributing to cardiac fibrosis [56, 57]. Thus, it is potential that EphrinB2 acts as a pivotal regulator in TGF-β-mediated organ fibrosis, as well as a promising therapeutic target.

#### EphrinB2 in human cardiac diseases

Up to now, the significant roles of EphrinB2 in human carcinogenesis have been clearly identified. High-level expression of EphrinB2 was associated with low-stage, progressive and metastable human solid tumors [58, 59], suggesting a certain therapeutic potential of EphrinB2 serving as a prognostic biomarker. Unlike the considerable achievements in clinical oncology, studies regarding on EphrinB2 in human cardiac diseases were scantly few. Lately, Levy et al. reported that a patient with a de novo variant in EFNB2 gene exhibited hypoplastic left ventricle and mild developmental delay [60]. EFNB2 mapped to 13q33.3 was located in the subtelomeric region of 13q chromosome. Recent cases have reported that patients with terminal deletion of 13q have developmental retardation and distinct congenital heart defects including tetralogy of fallot, ventricular septal defect, etc. EFNB2 was highly suspected as a candidate gene in these cases [61–63]. In another case with a familiar 13q33.2q33.3 deletion which was the smallest interstitial 13q33.3 deletion reported so far, only encompassing EFNB2 and ARGLU1, the patients exhibited congenital heart defects and neurological anomalies [60]. Levy et al. suggested that haploinsufficiency of the EFNB2 largely contributed to these developmental anomalies in heart and neural systems [60]. However, to make more precise phenotype-genotype correlations, further investigations with high-resolution SNP array data are encouraged.

### Potential therapies targeting EphrinB2

#### Proteases cleaving EphrinBs

Several proteases can cleave EphrinBs from their extracellular, transmembrane and intracellular domains in a manner that can be dispensable of Eph interaction. Protease of ADAM family, especially ADAM10, has been confirmed to cleave EphrinB2 extracellularly [29], while ADAM8 and ADAM13 were proved to cleave other EphrinBs [64, 65]. Besides, extracellular cleavage of EphrinBs by matrix metalloproteases (MMPs) was reported. The cleavage could be enhanced by the interaction with EphBs such as the case of EphrinB1 cleaved by MMP8 [64, 66]. These proteolytic fragments from ectodomain of EphrinBs preserved the binding activity to EphBs, regulating embryonic development, angiogenesis and fibrosis. In addition, EphrinBs were also the substrates of a rhomboid transmembrane serine protease-RHBDL2, which cleaved the transmembrane segment of EphrinBs, with a preference for EphrinB3. Such interaction eventually led to the inactivation and degradation of EphrinB3 [67]. In contrast, the intracellular domain of EphrinB2 cleaved by γ-secretase exhibited an enhancing role on the reverse signaling through phosphorylation of the down-stream molecules-Src and FAK [68, 69]. Thus, protease regulation of EphrinBs has broad significance for development and diseases. Blockade of specific protease may be a useful approach to inhibit aberrant EphrinBs function.

### Recombinant extracellular domain and antibodies

Recombinant extracellular domain (ECD) of Eph/ephrins were widely used in research, serving as soluble surrogates to activate as well as inhibit the bidirectional signaling [70]. The ECDs possessed high binding affinity to their counterparts. Coupling ECDs with Fc domain or albumin could further extend their half-life in vivo [71]. Numerous studies employing EphrinB2-Fc have illustrated its pro-angiogeneic role through activating EphB forward signaling [38, 72–74]. Meanwhile, a covalent coupling of EphrinB2 ECD with biomimetic fibrin vehicles could achieve the precise local delivery of EphrinB2. Such delivery managed to trigger angiogenic response locally, making it applicable in bioengineered tissues and therapeutic angiogenesis [75]. Recently, EphrinB2 ECD bio-conjugated with a soluble biopolymer and EphrinAs ECD embedded with nanoparticles were invented [76–78]. These nanoscaled technologies allowed an accurate modulation of Ephrin/Eph signalings in targeted cells, which has bright future in the emerging nanotherapy.

Although EphrinB2 ECD displayed as a soluble ligand activating EphBs, the soluble EphB4 ECD (sEphB4) [79], as well as its optimized form, sEphB4-HAS, which was fused of human serum albumin with sEphB4, blocked the EphrinB2/EphB4 bi-directional signaling. This blockage resulted in dysfunction of endothelium in-vitro and attenuation of pathological angiogenesis in vivo [80–82]. Furthermore, sEphB4 inhibited tumor invasion through blocking EphrinB2 [83]. Thus, sEphB4 could be utilized in anti-angiogenic therapy and the interference of EphrinB2 reverse signaling.

Additionally, inhibitory antibodies specifically targeting EphrinB2 and EphB4 have been applied in cancer research, showing high efficacy in inhibiting angiogenesis, lymphangiogenesis and tumor growth [84–86].

### Small molecules interrupting the interaction of EphrinB2 with EphB4

To date, small molecule targeting EphrinB-EphB interaction has been synthesized though with limited binding affinities. The low affinity could be blamed by the flexibility and large size of Ephrin-binding domain in EphBs [87]. Hence, more efficient but larger compounds (> 500 Da) were created to improve the affinity of EphrinB/EphB interaction. Koolpe, et al. identified a 15 amino acid-long peptide of~ 1700 Da named TNYL-RAW, which antagonized the EphrinB2/EphB4 interaction through specific binding to the Ephrin-binding pocket of EphB4 with low nanomolar affinity [88]. Later, several modifications were employed in TNYL-RAW. By fusion with the Fc portion of human IgG1 coupling to a polyethylene glycol (PEG) polymer, the PEGylated TNYL-RAW remarkably increased stability and half-life in vivo, making it practicable for in-vivo applications with long-term interference of EphrinB2/EphB4 interaction [89]. Meanwhile, another two smaller molecules of ~ 600-700 Da, synthesized from the C-terminal portion of the TNYL-RAW peptide were also reported which could inhibit EphrinB2 binding to EphB4 at low micromolar concentrations [90].

## Conclusion

EphrinB2 was a highly conserved protein ubiquitously expressed in all mammals, transducing signaling through a particular bidirectional pathway. EphrinB2-regulated bidirectional signaling was of great importance in cardiogenesis, guiding the completion of cardiac looping, expansion of endocardium, formation of myocardial trabeculation and cardiac valve and development of cardiac lineage differentiation as an emerging role. Lately, beyond cardiogenesis, EphrinB2 was identified critically involved in pathological cardiac remodeling process, such as angiogenesis after cardiac injury and cardiac fibrosis, which uncovered the tip of the iceberg regarding the roles of EphrinB2 under pathological conditions. Several proteases which were naturally existed in mammals, such as MMPs, ADAMs, γ-secretase, were capable of cleaving EphrinBs, leading to (de) activation of EphrinBs. Besides, synthesized recombinant ECD of Ephrin or Eph, inhibitory antibodies and small molecules were also able to (de) activate EphrinB2 signaling, providing a strongly potential target for further translational application in cardiovascular diseases.

## Abbreviations

ADAM: A Disintegrin And Metalloprotease
ECD: Recombinant extracellular domain
Eph: Erythropoietin-producing hatocellular receptors
Ephrin: Erythropoietin-producing hepatocellular receptor interacting proteins
ESC: Embryonic stem cell
hiPS: Human induced pluripotent stem cells
I/R: Ischemia/reperfusion
MI: Myocardial infarction
TGF-β: Transforming Growth Factor-β
